# Surface and Airborne Arsenic Concentrations in a Recreational Site near Las Vegas, Nevada, USA

**DOI:** 10.1371/journal.pone.0124271

**Published:** 2015-04-21

**Authors:** Dirk Goossens, Brenda J. Buck, Yuanxin Teng, Brett T. McLaurin

**Affiliations:** 1 Department of Geoscience, University of Nevada Las Vegas, Las Vegas, Nevada, United States of America; 2 Geography Research Group, Department of Earth and Environmental Sciences, KU Leuven, Leuven, Belgium; 3 Department of Environmental, Geographical and Geological Sciences, Bloomsburg University of Pennsylvania, Bloomsburg, Pennsylvania, United States of America; Institute for Health & the Environment, UNITED STATES

## Abstract

Elevated concentrations of arsenic, up to 7058 μg g^-1^ in topsoil and bedrock, and more than 0.03 μg m^-3^ in air on a 2-week basis, were measured in the Nellis Dunes Recreation Area (NDRA), a very popular off-road area near Las Vegas, Nevada, USA. The elevated arsenic concentrations in the topsoil and bedrock are correlated to outcrops of yellow sandstone belonging to the Muddy Creek Formation (≈ 10 to 4 Ma) and to faults crossing the area. Mineralized fluids moved to the surface through the faults and deposited the arsenic. A technique was developed to calculate airborne arsenic concentrations from the arsenic content in the topsoil. The technique was tested by comparing calculated with measured concentrations at 34 locations in the NDRA, for 3 periods of 2 weeks each. We then applied it to calculate airborne arsenic concentrations for more than 500 locations all over the NDRA. The highest airborne arsenic concentrations occur over sand dunes and other zones with a surficial layer of aeolian sand. Ironically these areas show the lowest levels of arsenic in the topsoil. However, they are highly susceptible to wind erosion and emit very large amounts of sand and dust during episodes of strong winds, thereby also emitting much arsenic. Elsewhere in the NDRA, in areas not or only very slightly affected by wind erosion, airborne arsenic levels equal the background level for airborne arsenic in the USA, approximately 0.0004 μg m^-3^. The results of this study are important because the NDRA is visited by more than 300,000 people annually.

## Introduction

Increased global interest in the role of mineral dust in human disease is occurring due to climate change, desertification, and expansion of populations into increasingly more arid landscapes [1]. Natural and anthropogenic activities in arid landscapes generate significant dust emissions, which may expose populations to potentially hazardous substances such as heavy metals, infectious agents, naturally occurring asbestos, or silica [2–5].

**Fig 1:**
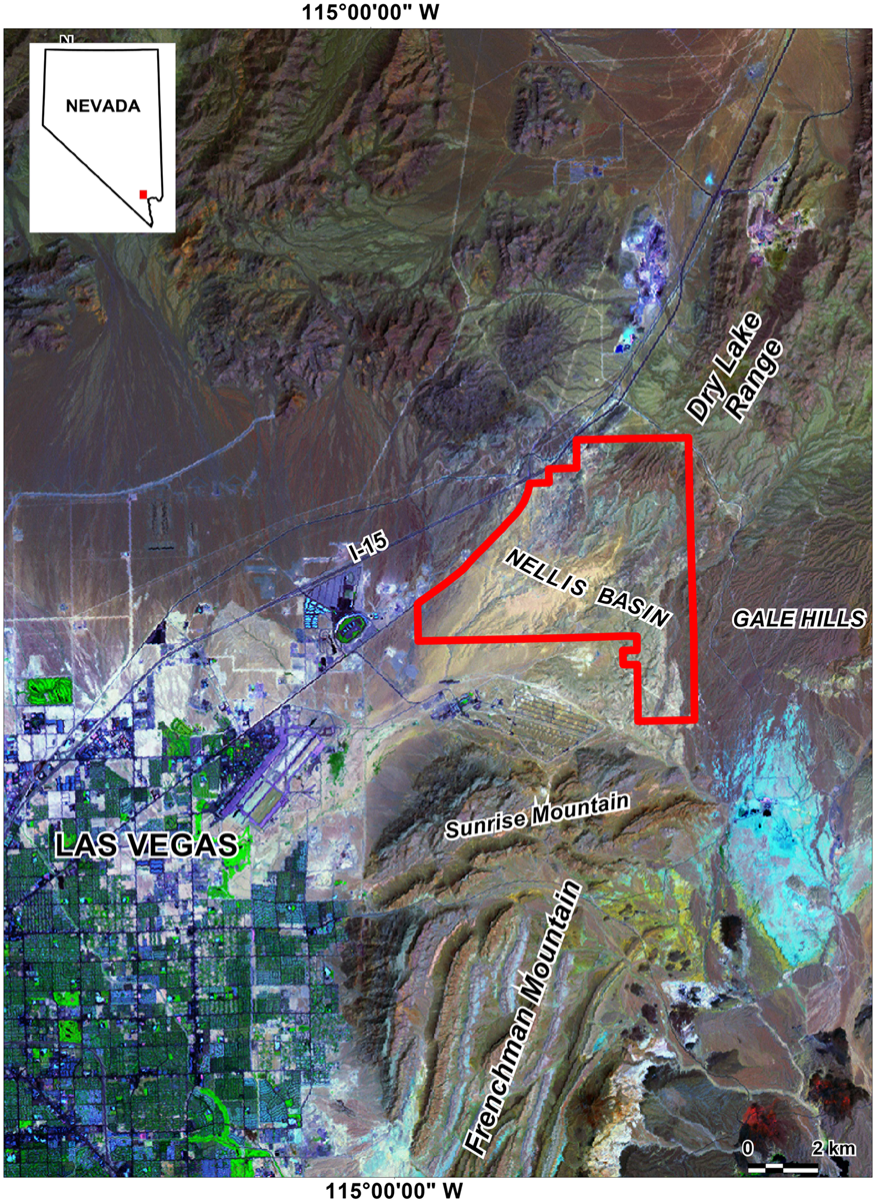
Study area location map. Satellite image from the NASA Landsat Program (NASA Landsat Program, 2000, Landsat ETM+ scene p039r035_7t20000503_z11, U.S. Geological Survey, Sioux Falls, 5/3/2000)

In a previous study [6] elevated concentrations of naturally occurring arsenic were detected in the Nellis Dunes Recreation Area (NDRA), a 36 km^2^ area located just six kilometers northeast of the metropolitan area of Las Vegas, North Las Vegas and Henderson, Nevada, USA (Fig 1). Concentrations up to 83 μg g^-1^ were measured in soil (upper 0–3 cm), and up to 312 μg g^-1^ in airborne dust. For comparison, the world’s average arsenic concentration in soil is of the order of only 5 to 7 μg g^-1^ [7,8]. The arsenic in the NDRA is particularly abundant in a yellow colored sandstone belonging to part of the Muddy Creek Formation (≈ 10 to 4 Ma), which occurs over 20.2% of the NDRA. In addition, wind erosion has deposited a nearly continuous layer of windblown sand from this geologic unit over another 14.1% of the NDRA. In the south-central part of the area the windblown sand is so abundant that it has formed up to 6 m thick reversing dunes that are active all year. Previous research [9,10] has shown that all sand-covered areas in the NDRA, including the dunes, are very emissive, producing up to 2 t ha^-1^ (<20 μm fraction), another 2 t ha^-1^ (20–60 μm fraction), and even up to 20 t ha^-1^ (60–100 μm fraction) of windblown particles annually. Water erosion from the areas with yellow sand has also transported much arsenic to the washes. After drying, these washes then transform into secondary sources of windblown arsenic [6].

Apart from wind erosion, much arsenic is also emitted in the NDRA by human activities. The most important activity is off-road vehicular driving (ORV). According to a Bureau of Land Management study [11], nearly 300,000 people visit the NDRA annually to drive their vehicles in the dunes, washes, desert pavements, rock-covered hills and moon-like landscapes that characterize this part of the Mojave Desert. This number is still conservative because the number of off-road drivers in southern Nevada has almost quadrupled since the year 2000 [12]. Studies by Goossens and Buck [13] and Goossens et al. [10] have shown that, in the NDRA, the total amount of dust emitted by ORV activities is comparable to that emitted by wind erosion. For the year 2008, the annual numbers were 1794 tons (wind erosion) and 1711 tons (ORV). Much of this dust was emitted from areas rich in arsenic. Therefore, emission of arsenic is of concern to the NDRA, especially because of the large populations visiting the area.

To investigate the arsenic in the NDRA in more detail, samples from a previous study [9] were re-analyzed and combined with new information. This article presents some of the results. The aims of the study are: (1) document the spatial occurrence of arsenic in the soil (upper 0–4 cm) in the NDRA; (2) study the airborne arsenic concentrations at various altitudes above the ground; and (3) investigate the spatial distribution of the airborne arsenic over the NDRA. This information is used to provide data on exposure scenarios as part of a human health risk assessment for the area. Additional topics investigated were: arsenic speciation, bioaccessibility, bioavailability, and other toxicological assessments related to dust exposure at NDRA. These topics will be dealt with in subsequent publications.

## Study Area

**Fig 2:**
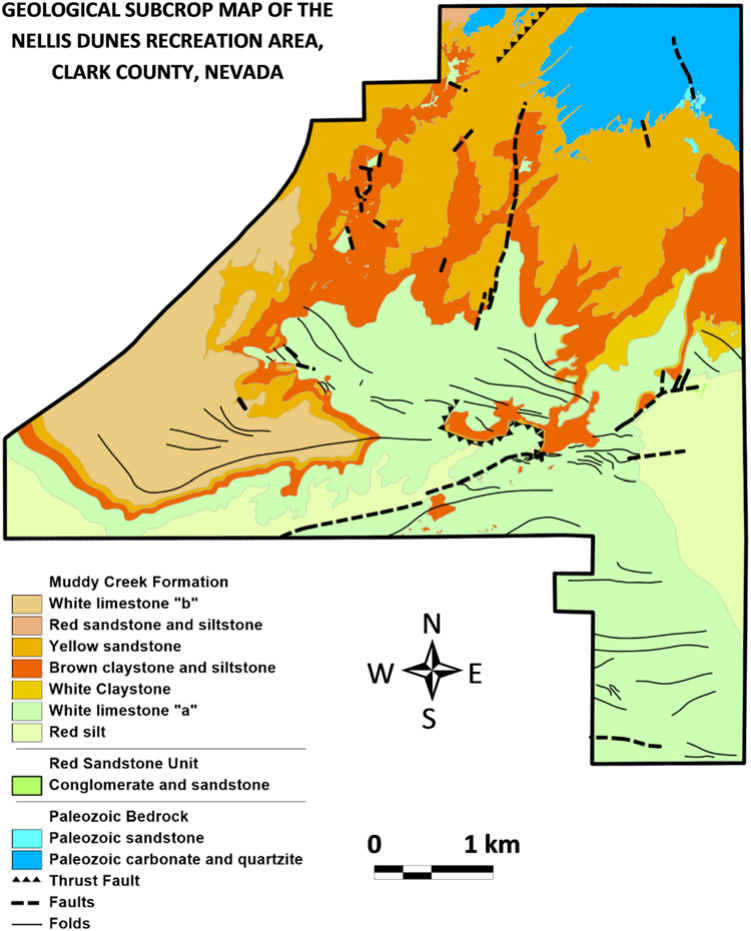
Subcrop geologic map of the Nellis Dunes Recreation Area.

Nellis Dunes Recreation Area is located on the eastern side of the Las Vegas Valley, Nevada, USA, between the Las Vegas and Dry Lake Ranges (to the N) and the Sunrise and Frenchman Mountains (to the S). It is primarily composed of incised fan remnants and exposed Neogene and Quaternary sediments, except for the mountains in the northeast, which are Paleozoic and composed predominantly of limestone [13]. The Neogene deposits within the field area are assigned to the Muddy Creek Formation (≈ 10 to 4 Ma) [14,15]. They consist of a red silt facies covered by a 5–50 m thick white limestone, which may contain a marlish zone near its base. Above the limestone occurs a brown clay/silt unit several meters thick. This silt is covered by yellow sand and sandstone (called the yellow sandstone unit in this study) that contains most of the arsenic. The yellow sandstone unit can be several tens of meters thick and locally contains zones of white, bleached sandstone. It crops out in much of the northern part of the NDRA, or is covered by only a thin (several cm) surficial layer of recent dust and dispersed Quaternary gravel clasts. In the NW corner of the NDRA the yellow sand is covered by red silt and sand, and near the western border, by a second limestone unit. Fig 2 is a geologic map of the area.

Apart from the yellow sand, elevated arsenic concentrations also occur near faults, on the contact between the white limestone and the brown silt [16].

Neogene to Quaternary fan remnants and inset fans occur throughout the field area. These alluvial gravels are capped by extensive petrocalcic horizons and overlie the Muddy Creek Formation. Extensive incision, particularly in the northern portion of the field area, has exposed the fine-grained Muddy Creek Formation. The middle of the southern portion of the field area is occupied by an extensive zone of dune sands, which cover the Neogene deposits. Although much of the sand is generally less than a meter thick, many highly active reversing dunes (oriented NW—SE) are present. These dunes may be up to 250 m long and are among the most popular off-road driving zones within the area [13].

Soil development is negligible in the active sand dunes and areas where bedrock is exposed, including the badlands of the exposed Muddy Creek Formation. Surficial characteristics in these regions are controlled by the underlying bedrock geology or dune sand characteristics. In the remaining areas, primarily the fan remnants, the soils are characterized by thin (0–5 cm), platy, alkaline, Av (vesicular) horizons containing low amounts of organic matter overlying calcic and/or petrocalcic horizons. Vesicular A (Av) horizons are generally associated with desert pavements. In many areas, particularly in the western portion of the field area, the surface horizons are eroded and petrocalcic horizons are exposed at the surface. Most of the surface gravels are composed of broken fragments of the petrocalcic horizons in these areas. Soils in the study area are classified as Typic Haplocalcids, Calcic Petrocalcids, and Typic Torriorthents [13].

**Fig 3:**
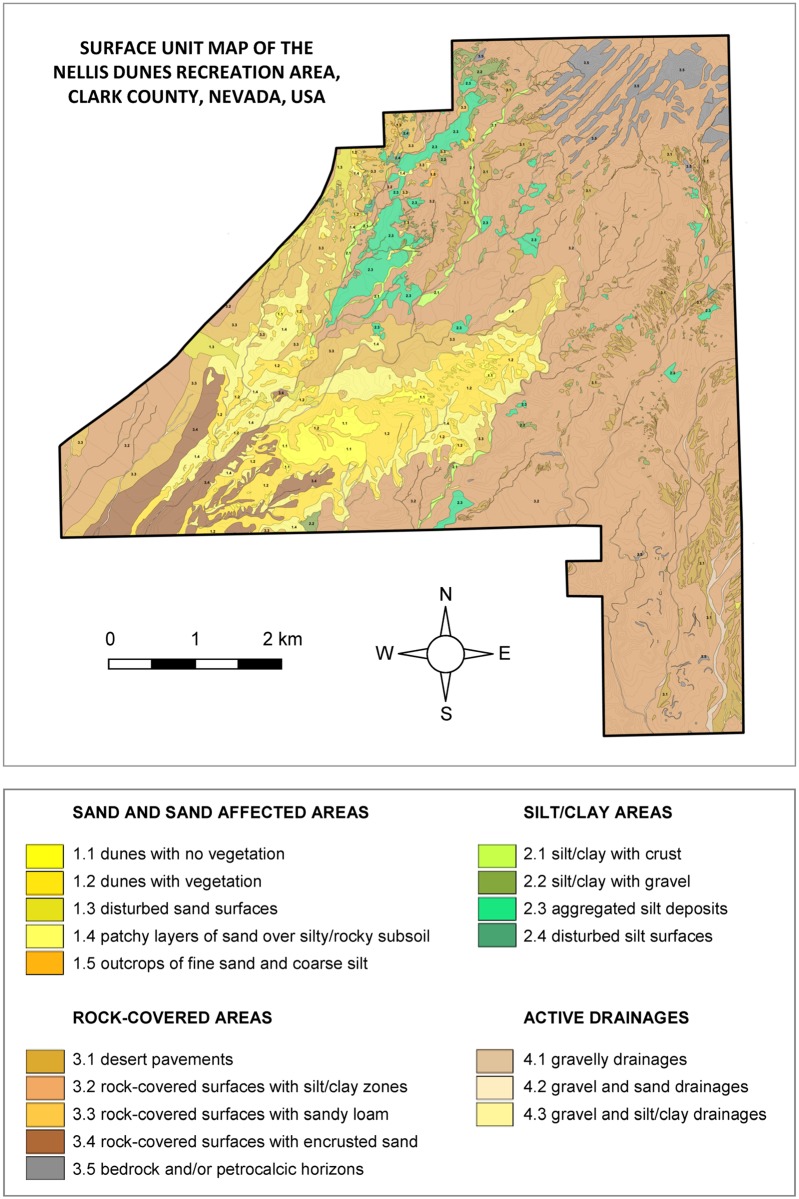
Occurrence of the 17 surface units at the Nellis Dunes Recreation Area.

Previous studies of dust emissions in the NDRA [13,17,18] identified and mapped four major surface classes within the study area comprising a total of 17 types of surfaces (Fig 3). These surface types were identified based on their potential to emit dust (see [18] for information on the criteria used). A description of each unit is provided in Table 1; photos of the units can be consulted in [13]. The major surface classes include:

1. Sands and sand-affected areas: active or stabilized sands, with or without rock fragments and/or vegetation.
2. Silt/clay areas: loose and slightly stabilized silt/clay deposits, with or without sparse rock fragments exposed at the surface.
3. Rock-covered areas: stabilized silty or sandy silty deposits with numerous rock fragments on top, desert pavements over a silty sublayer, bedrock, and petrocalcic horizons.
4. Active drainages: active drainages in sand and silt areas, and gravelly drainages.

**Table 1:** Overview and characteristics of the 17 surface units in the Nellis Dunes Recreation Area.

Map unit	Description	Rock fragments	Surface crust	Vegetation
*Sand and sand-affected areas*				
1.1	Active dunes without vegetation. Decimeter to several meters thick.	Sparse; may have exposed petrocalcic horizons	Absent	Absent
1.2	Active dunes with vegetation. Coppice dunes <50 cm may be present.	Sparse; <5% rock cover	Absent	Isolated shrubs
1.3	Anthropogenic disturbed sand surfaces. Typically <2–3 cm thick loose sands overlying petrocalcic horizons or bedrock.	Common, mixed with 2–3 cm thick loose sans overlying bedrock	Absent	Absent
1.4	Patchy, shallow (1–3 cm thick), loose sand overlying silty/rocky subsoil	Common, not interlocking, rocks in subsoil are exposed at surface	Absent	Isolated shrubs
1.5	Very fine sand and coarse silt outcrops. Commonly badlands.	Absent	Physical	Mostly absent
*Silt/clay areas*				
2.1	Silt/clay outcrops with biological crust	Sparse, <3–4% rock cover	Biologic	Isolated shrubs
2.2	Silt/clay outcrops with gravel	Common, <15%, not interlocking	Physical	Usually absent
2.3	Aggregated silt deposits, commonly badlands, aggregates <5 mm diameter	Absent	Physical, patchy	Absent
2.4	Anthropogenic disturbed silt surfaces	Variable, not interlocking	Absent	Absent
*Rock-covered areas*				
3.1	Well-developed desert pavements with underlying silty Av horizon	Abundant: tightly interlocking rock fragments, nearly 100% surface cover	Physical between rock fragments	Rare, isolated shrubs
3.2	Rock-covered surface with silt/clay	Many: 60–80%, poorly interlocking	Physical and biological between rock fragments	Common, shrubs (10–15%)
3.3	Rock-covered surface with sandy loam	Many: 60–80%, poorly interlocking	Physical and biological between rock fragments	Common, shrubs (10–15%)
3.4	Rock-covered with encrusted sand and biological crusts	Common: 20–30%, poorly interlocking	Biological, continuous	Common, shrubs (10%)
3.5	Bedrock and/or exposed petrocalcic horizons	Continuous rock outcrop	Absent	Rare shrubs
*Active drainages*				
4.1	Gravelly drainages, without fine sediment	Abundant: 90–100%, non-interlocking gravel clasts	Absent	Absent
4.2	Gravel and sand drainages	Abundant: 70–80% with sand mixture	Absent	Absent
4.3	Gravel and silt/clay drainages	Common: 30–60%, poorly interlocking, with silt mixture	Physical	Common, shrubs (10–30%)

## Methods and Procedure

### Strategy

Because of the spatial complexity of NDRA in regards to geology, soil, topography and dust emissions, it is imperative to collect data from a very large number of locations. This can be easily done for soil samples. For airborne dust it is much more difficult, for two reasons: (1) the prohibitive cost for airborne sample collection and subsequent laboratory analysis; (2) the significant risk of vandalism for sensitive and expensive equipment, as we learned from previous field work in the area. Therefore, for airborne dust, the following strategy was adopted:

1. Airborne dust samples were collected from 2-m long poles equipped with passive dust samplers (see section Field Procedure for more information). Permission to install these poles was granted by the Las Vegas office of the Bureau of Land Management, who manages the NDRA. There were 68 poles in total, 4 poles for each surface unit. For practical reasons the poles were installed in 34 groups ("dust stations") of two poles each. In each dust station the poles were installed in the same area, between 5 and 20 m from each other. There was no interference between the poles and each pole can be considered an independent site, measuring its own dust dynamics. Since the four poles belong to a same surface unit and the units were selected based on dust emission potential, we can expect that the emission (and the airborne concentrations resulting from the emission) are similar for these four locations. Routine checks confirmed that the differences were indeed small, usually within 25%. The average emission and concentrations were then used for each surface unit in the calculations.
2. Surface samples were taken from 570 locations throughout the NDRA. These locations included the dust stations where the airborne dust was collected. For the 34 dust stations sediment was thus available for both soil and airborne dust, which allowed us to compare the arsenic content in the soil with the arsenic content in the air. Permission for sampling was granted by the Las Vegas office of the Bureau of Land Management.
3. By establishing the relationship (if any) between the arsenic content in the airborne sample and the arsenic content in the soil sample, and assuming that the airborne dust at a dust station predominantly originates from local erosion (see further in this section), the arsenic content in the airborne sample at a given location can be estimated based on the arsenic content in the topsoil from which the airborne dust was eroded. One should realize that the existence of a relationship between the arsenic content in an airborne sample and the arsenic content of a soil sample is not trivial, for import of airborne dust from other (distant) sources may contaminate the locally collected airborne sample. We will investigate later in this article whether any relationship exists, and if one exists, how it can be characterized.
4. Assuming that the arsenic content of airborne dust at a particular location can be estimated from the arsenic content of the topsoil, we still don't know the airborne arsenic concentration (expressed in arsenic weight per volume of air, for example, in μg m^-3^). To derive that information we first need to know the airborne dust concentration. This is not a problem at the 68 sampling poles, where the airborne dust concentrations were directly measured. To derive the airborne dust concentrations for locations without dust poles the following procedure can be followed. First, since the airborne dust is a direct result from aeolian emission, we investigate if any relationship exists between the aeolian dust emission rate (expressed in, for example, g m^-2^ s^-1^) and the airborne dust concentration (g cm^-3^). Note that, if such a relationship exists, it will depend on elevation (height above the surface). Accepting that there is a relationship, and further accepting that the emission rate is identical for all locations belonging to a same surface unit (which is, of course, an approximation, but which also makes sense because the surface units were selected based on emission criteria), we can reconstruct the airborne dust concentrations at a given location provided the correct surface unit for that location is known. We acknowledge that the result is only an estimate, but the order of magnitude should be correct.
5. By combining the arsenic content in the airborne dust sample with the airborne dust concentration the airborne arsenic concentration can then be calculated.

Although logical, whether or not the procedure outlined above is successful and will lead to at least acceptable results depends on the strength of the relationships (a) between the arsenic content in the airborne sample and the one in the soil, and (b) between the airborne dust concentration and dust emission, if these relationships exist at all. The validity of these assumptions is tested in the section Test of the Method.

### Field Procedure

All dust samples were collected with passive Big Spring Number Eight (BSNE) dust samplers (see [19] for technical details on the sampler). We used BSNEs because this sampler is widely used in dust research, facilitating comparisons with other studies, and also because its collection efficiency is known for a wide range of particle sizes and wind speeds (see [20] for an overview of available literature). Four BSNEs were installed on each pole, at 25 cm, 50 cm, 75 cm and 100 cm above the local surface.

**Fig 4:**
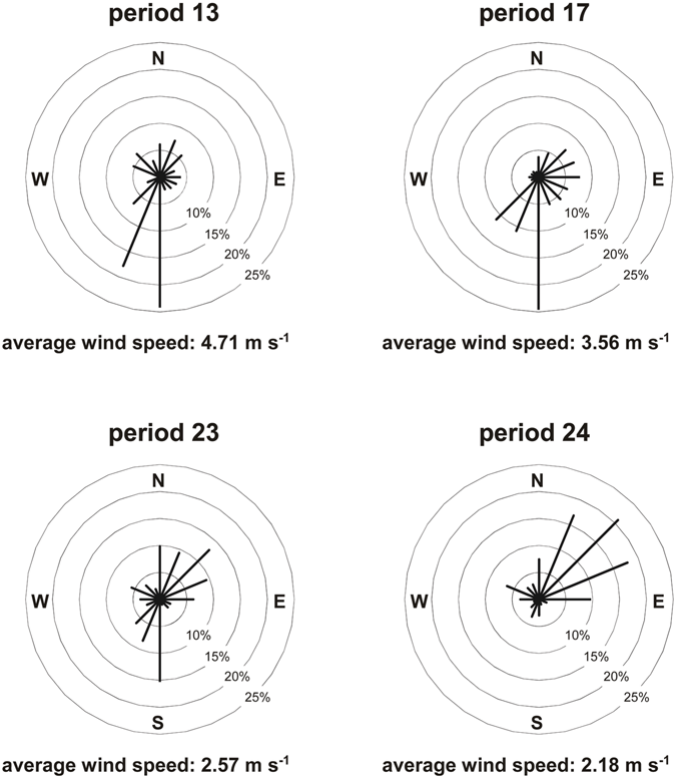
Average wind speed and wind rose for the four periods tested in this study. Period 13: 30 May 2008–12 June 2008; Period 17: 29 July 2008–14 August 2008; Period 23: 21 October 2008–4 November 2008; Period 24: 4 November 2008–18 November 2008.

Dust was collected after every two weeks. Originally 26 two-week periods were sampled, but most of the samples were consumed during analyses carried out for a previous project. Enough sample was left for four two-week periods (period 13: 30 May 2008–12 June 2008; period 17: 29 July 2008–14 August 2008; period 23: 21 October 2008–4 November 2008; period 24: 4 November 2008–18 November 2008), and these samples were used in the current study. Although the number of periods is limited to only four, they adequately span the range of dust emissions observed during the original 26 periods. One of the periods was very emissive, the second moderately emissive, the third slightly emissive, and the fourth had only very low emissions. Average wind speed and a wind rose are shown for each period in Fig 4. Substantial differences were measured between the periods, which means that the arsenic and dust data in this study include the natural variability in wind regime in the test area and are not just replicas of the same condition. Although we recognize that four periods is not the ideal situation we didn't observe climate conditions in the other time periods of sufficient severity to suggest that these four periods were not typical of the broader time period of one year considered. To ensure objectivity, we keep the four periods as individual study objects in the analysis and refrain from averaging the data (to reconstruct, for example, annual patterns).

Three 20-m wind towers and one 10-m wind tower were erected in the NDRA to measure the wind speed, necessary to calculate the airborne dust concentrations. Each cluster of dust stations contained at least one wind tower. Additional wind measurements were performed at all dust stations with a portable 3-m long wind tower containing four anemometers (heights: 56 cm, 121 cm, 202 cm and 259 cm) to determine the roughness length and the wind profile near each pole. In total, 396 periods of 10 minutes each were sampled with the portable tower. Wind speeds at all catcher levels (25 cm, 50 cm, 75 cm and 100 cm) were then calculated and linked to the data collected from the four wind towers. For a more detailed description of the procedure and a test of the method we refer to the study by Goossens and Buck [9], p. 100.

### Laboratory Analysis

All airborne (BSNE) and soil samples were air dried upon arrival in the laboratory. Soil samples were then sieved in a HEPA filtered hood using plastic sieves to remove coarse fragments (> 2 mm). No such sieving was necessary for the airborne samples, which did not contain coarse fragments. Samples were then digested in accordance with the USGS Four-Acids Method [21] and subsequently analyzed for chemical composition using an Agilent 7700 inductively coupled plasma / mass spectrometry (ICP-MS) analyzer (Agilent Technologies, Santa Clara, USA). To ensure quality control for the ICP-MS analyses, all quality control procedures set forth by EPA Method 6020A [22] were conscientiously followed. NIST SRM 8704 (Buffalo River Sediment) and NIST SRM 2711a (Montana II Soil) were used as standard reference materials. All analyses passed the EPA Method 6020A limits.

### Dust Emission Rate

For the dust emission rate at each dust station we used the data from [23]. Although the method adopted to calculate the emission requires the input of airborne concentration data it does not preclude one from objectively checking a potential relationship between dust emission and dust concentration because it is based on particle exchange theory and thus only considers vertical gradients of concentration (not absolute concentrations).

## Test of the Method

To test whether or not the technique outlined above (see section Strategy) works we applied it to the 34 dust stations, for which the actual airborne arsenic concentrations are known. We tested the technique for five cases: calculation of the airborne arsenic concentrations at 25 cm, 50 cm, 75 cm and 100 cm above the ground, and the average (based on vertical integration) airborne arsenic concentration in the lowermost meter. Here we show the test for the latter case; results for the distinct altitudes were similar.

### Step 1: Relationship between Arsenic Content of Airborne Dust and Arsenic Content of Topsoil

**Fig 5:**
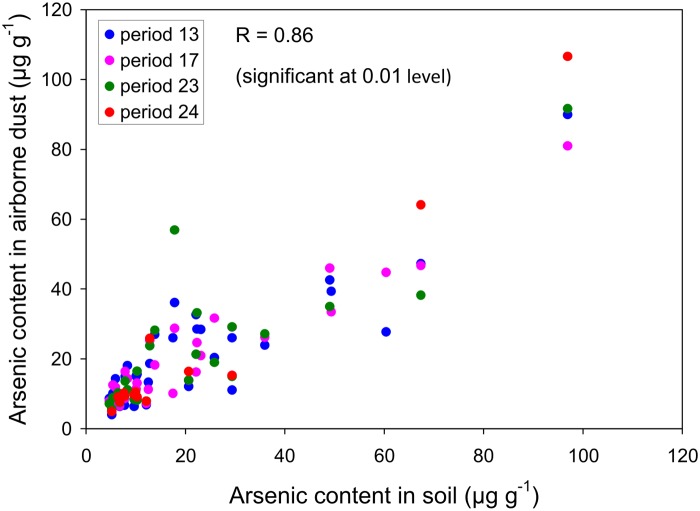
Arsenic content in airborne dust (average value for first meter above the ground) *versus* arsenic content in topsoil (fraction <2 mm).

The average airborne arsenic content in the lowermost meter (based on vertical integration, see above) was plotted against the arsenic content in the topsoil (Fig 5). Apart from one outlier in period 23 the pattern is consistent and shows that the arsenic content in airborne dust was strongly correlated to the arsenic content in the topsoil. This confirms that the dust collected by the BSNEs is predominantly local dust, eroded from nearby the sampling area. The effect of dust from distant sources is limited because such dust will be rather fine and should only slightly contribute to the total dust load in the BSNEs. However, it might be responsible for at least a portion of the spread in the data. Although the relationship in Fig 5 approaches a 1:1 relationship, this may differ according to the height at which the airborne samples are taken. For this study the relationships were as follows (average for the four periods investigated): 0.8:1 at 100 cm, 0.9:1 at 75 cm, 1.0:1 at 50 cm, and 1.0:1 at 25 cm. This shows that the influence of remotely eroded dust diminishes the closer the local surface is approached (as can be expected), and remains negligibly small in the first 50 cm above the ground, at least in this study.

Not all four periods were equally emissive (see section Field Procedure), and therefore, the grain size of the dust collected in each BSNE slightly differed between periods (see [9], p. 117 for details). Because the arsenic content is usually higher in the finest fractions [6] the relationship shown in Fig 5 may therefore also differ between periods, although the data indicate that these differences remain rather small. To ensure correct calculations, we used the relationship for each period separately when calculating the airborne arsenic concentrations for that specific period.

### Step 2: Relationship between Dust Emission and Airborne Dust Concentration

**Fig 6:**
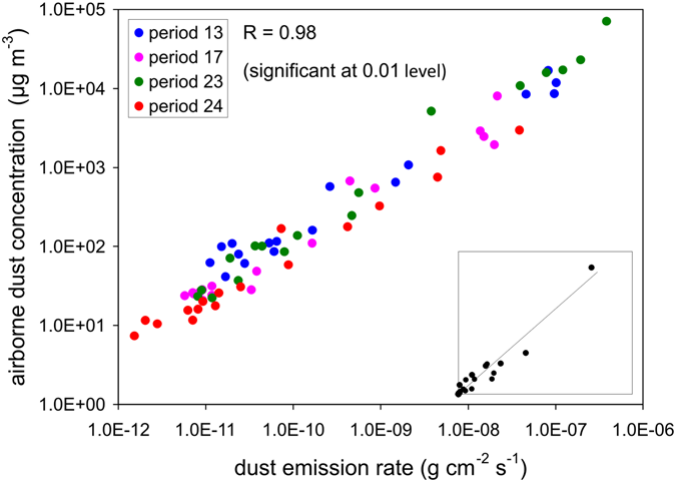
Dust emission rate *versus* airborne dust concentration. The inset shows the same data with linear scales.

Since our ultimate goal is to reconstruct the airborne arsenic concentration for any arbitrary location in the NDRA we need to work with emission rates based on surface units. The emission rates and airborne dust concentrations were plotted in Fig 6 for all 17 surface units, for all 4 periods. Recall that the airborne concentrations in Fig 6 are the average concentrations (based on vertical integration) in the first 1 m above the ground; but the patterns were identical for the four elevations separately (25, 50, 75 and 100 cm). Also note that, to allow correct comparisons, the emission and concentration data were calculated for identical dust size fractions (in this case: total suspended dust or TSP, i.e., all dust collected by the BSNEs).

A strong linear relationship exists between emission and airborne concentration (Fig 6). Since the range of emission rates was very large, more than five orders of magnitude, logarithmic scales had to be used in the main diagram in Fig 6 to adequately display all the data points. The inset shows the data with linear scales, confirming the linear character of the relationship. Similar to the relationship between the arsenic content in the soil and the arsenic content in the airborne dust (Fig 5), the relationship between dust emission and airborne dust concentration may slightly vary between periods, but the differences are small (Fig 6). This was also confirmed for each of the distinct heights of 25, 50, 75 and 100 cm.

### Step 3: Reconstruction of the Airborne Arsenic Concentration

To calculate the airborne arsenic concentration (mass of arsenic per volume of air) the arsenic content in the airborne dust should be multiplied by the airborne dust concentration.

### Example

**Fig 7:**
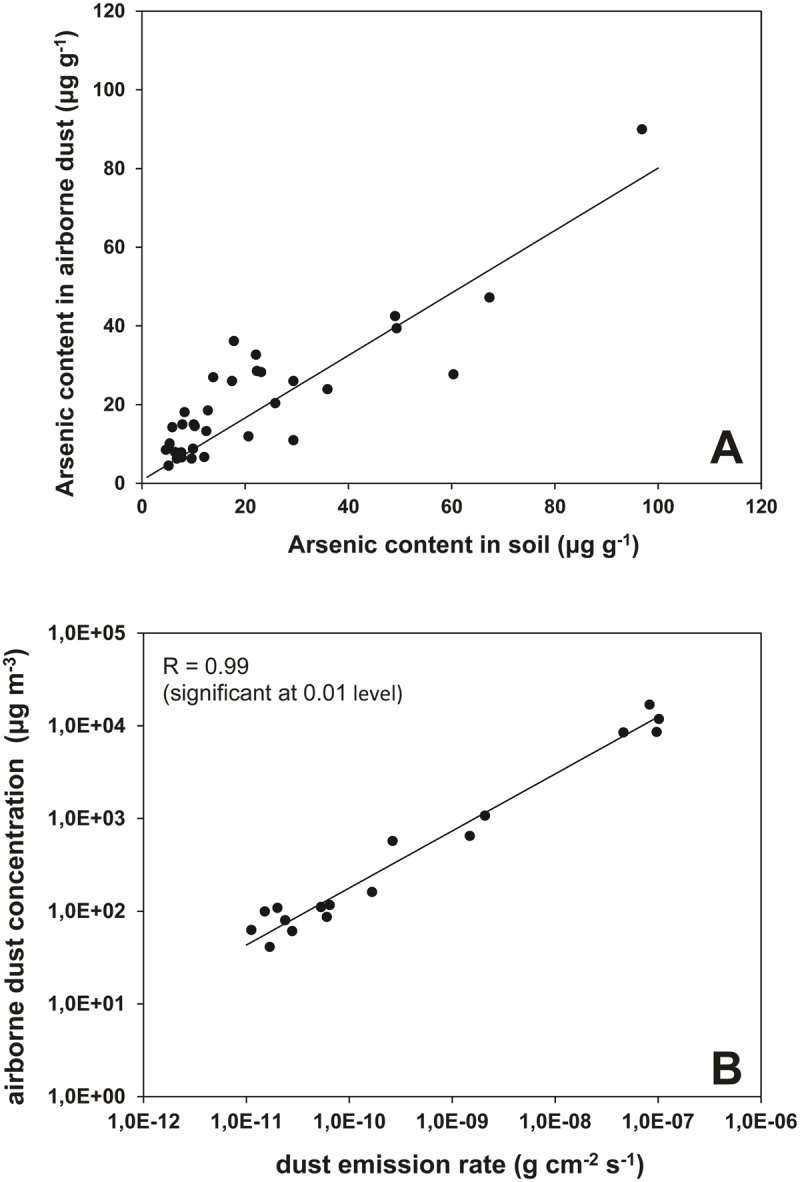
Relationships for experimental period 13. A: arsenic content in airborne dust *versus* arsenic content in topsoil; B: dust emission rate *versus* airborne dust concentration.

To illustrate the technique we calculate the airborne arsenic concentrations for periods 17, 23 and 24 solely based on the data from period 13. The relationships between the measured arsenic content in the soil and the measured arsenic content in the airborne dust (Fig 7A) and between measured dust concentration and dust emission (Fig 7B) that are used in the calculations are thus only those for period 13; this ensures a 100% independent calculation of the data for the other three periods.

**Fig 8:**
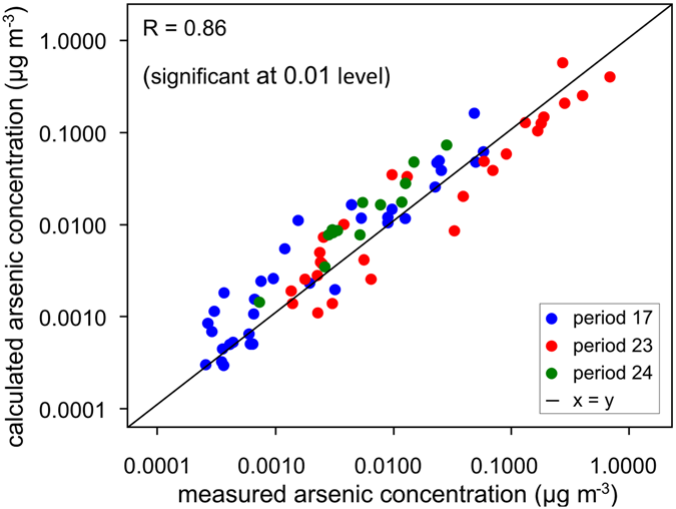
Calculated and measured airborne arsenic concentrations for experimental periods 17, 23 and 24.

The result is presented in Fig 8, where calculated arsenic concentrations are compared to measured arsenic concentrations. Although logarithmic scales had to be used to adequately display all the data points, the data match well, showing that the technique leads to acceptable results. Grouping the data into concentration classes, which is necessary to produce arsenic distribution maps, further reduces the spread in the result. Analogous tests for 25, 50, 75 and 100 cm also resulted in a good agreement between the calculated and measured concentrations, for all periods and dust stations.

### Airborne Arsenic Concentrations at Arbitrary Heights

The airborne arsenic concentration at any arbitrary height can be calculated by interpolation or extrapolation of the data. Of course, this only works well if concentration and height are sufficiently related to each other, and if extrapolation remains within reasonable limits.

**Fig 9:**
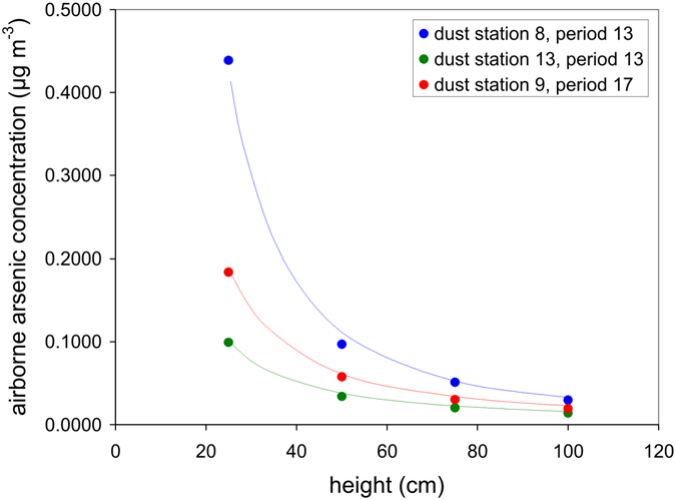
Airborne arsenic concentration as a function of height: 3 examples.

Analysis of case studies (Fig 9 presents only three of many more examples) shows that, at least in our study area, airborne arsenic concentration is strongly related to elevation above the surface according to a power law relationship *C* = *az* ^*b*^, where *C* is airborne arsenic concentration, *z* is elevation, and *a* and *b* are empirically derived numbers (*b* quantifies the rate of decrease of the arsenic concentration with height). Using the power law, *C* can be calculated at any desired height.

## Arsenic Maps for the Nellis Dunes Recreation Area

### Arsenic at the Surface (Topsoil and Bedrock)

#### Occurrence

**Fig 10:**
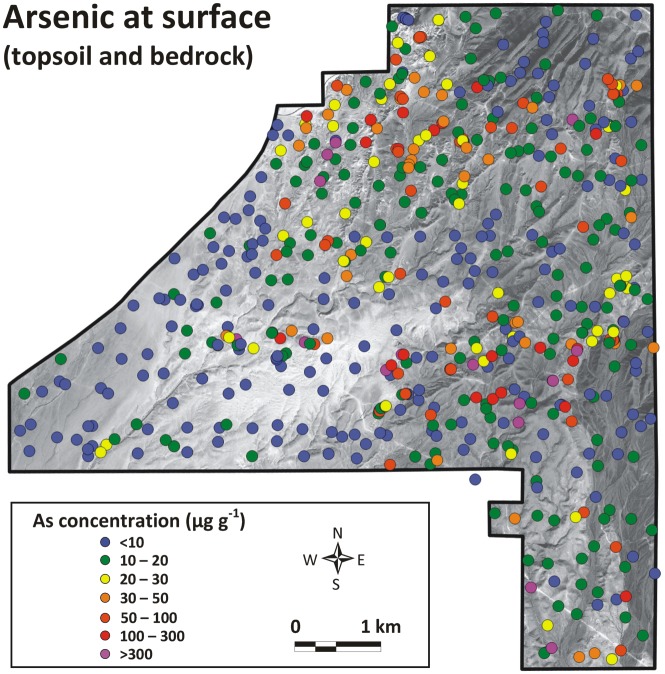
Arsenic concentration at the surface. A total of 570 points were sampled. (Background aerial photograph image: Copyright 2009 DigitalGlobe)

The arsenic content at the surface was measured at 570 locations in the NDRA (Fig 10). Four observations can be made:

1. The arsenic content at the surface varies considerably within the NDRA. Most locations have arsenic concentrations between 5 and 20 μg g^-1^, but there are many areas where the concentrations are much higher, even more than 1000 μg g^-1^. The highest value we measured was 7058 μg g^-1^. Therefore, many places in the Nellis Dunes area are very rich in arsenic.
2. There are three major zones in the NDRA where elevated arsenic concentrations occur. The first is located in the northwest, where the arsenic content is always >10 μg g^-1^, and in many cases >20 (or even >30) μg g^-1^. The second zone is a WNW-ESE strip in the center, with several places of arsenic concentrations >300 μg g^-1^, or locally even >1000 μg g^-1^. The third zone is in the extreme southeast, especially near the southern border of the NDRA. Arsenic concentrations up to 446 μg g^-1^ were measured in this area.
3. The lowest arsenic concentrations occur in the southwestern portion of the NDRA and in the dunes in the center. The arsenic content in the topsoil in these areas is almost always <10 μg g^-1^.
4. Elsewhere in the NDRA arsenic concentrations in the topsoil are of the order of 10–20 μg g^-1^, still above the world’s average of 5–7 μg g^-1^.

#### Relationship with geology

The occurrence of arsenic at the surface is strongly linked to the geological framework of the NDRA. Two geologic parameters are important: the presence of the yellow sandstone unit, and the presence of faults.

**Fig 11:**
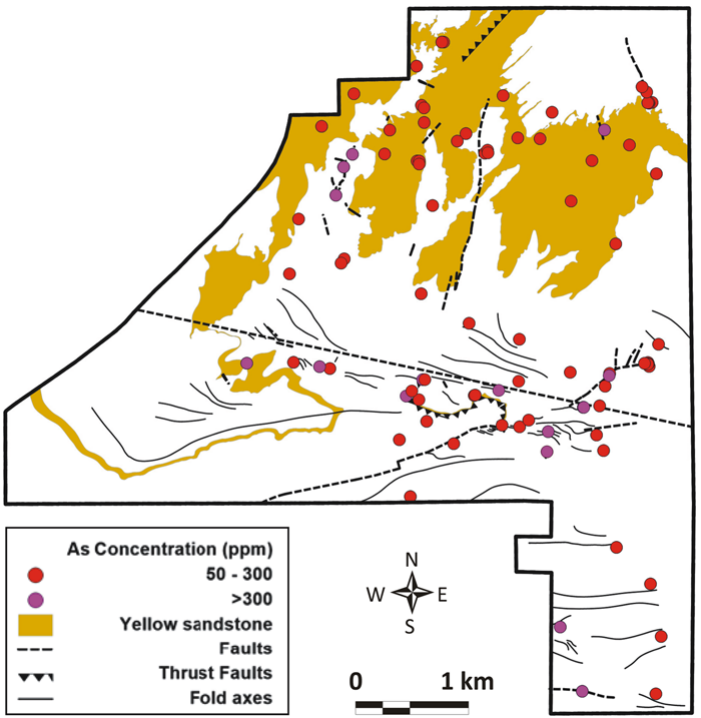
Location of arsenic rich sampling points (>50 μg g-1) and the occurrence of the yellow sandstone unit and tectonic faults.

Outcrops of the *yellow sandstone unit* explain most of the occurrences of high arsenic concentrations in the north of the NDRA. Fig 11 is an overlay of the locations with more than 50 μg g^-1^ arsenic at the surface and the occurrence of the yellow sandstone unit. The correlation between the patterns is clear. Note that at many places the yellow sandstone is covered by a thin (a few cm, up to maximum a few dm) surficial layer of recent Quaternary dust mixed with limestone pebbles eroded from the Paleozoic carbonate in the northeast corner of the NDRA. Here the arsenic levels are lower because of the absence of yellow sandstone outcrops. However, underneath the surficial Quaternary cover the arsenic levels are very high, comparable to those in the outcrops.

The *relationship between high arsenic concentrations and faults* is also shown in Fig 11. Only the major faults have been drawn in the figure; many local, much smaller faults occur in the NDRA, especially in the limestone in the southeast corner of the area. These secondary faults are too limited in lateral extent to be included in Fig 11, and their offset is also very limited, of the order of only a few centimeters to tens of centimeters. All occurrences of >300 μg g^-1^ of arsenic are located in the direct vicinity of the major faults: in the northwest, near the central fault system in the center, and in the far southeast. Mineralized fluids have moved through these faults, bringing the arsenic to the surface. Older faults in the Paleozoic carbonate in the northeast corner of the NDRA also mark zones with high arsenic concentrations (Fig 11).

#### Relationship with particle size

**Fig 12:**
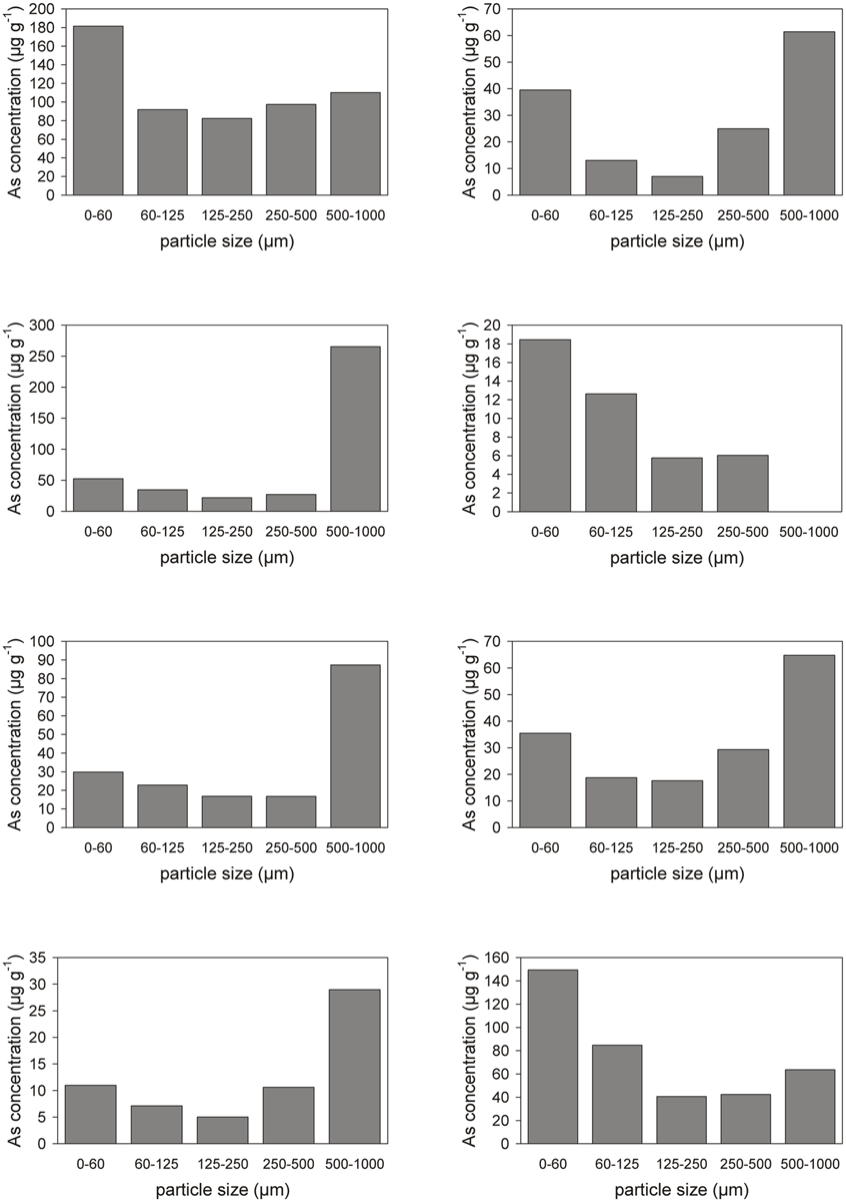
Arsenic concentration in different grain size fractions, for 8 topsoil samples taken from the NDRA.

The lowest arsenic concentrations (at the surface) occur in the southwest of the NDRA, near the western border, and in the center-south. All these areas are characterized by sandy topsoil: sand dunes in the center-south, and a thin (1–3 cm) sometimes discontinuous layer of dune sand in the southwest and west. The correlation between low levels of arsenic and coarse sand deposits is very prominent, as can be seen by comparing Figs 3 and 10. To investigate this further we sieved eight topsoil samples, all from different surface units and different textural compositions, into different size fractions and measured the arsenic concentration in these fractions. Results (Fig 12) show a consistent trend, with the lowest arsenic concentrations in the fraction 125–250 μm and higher arsenic concentrations as the sediment becomes coarser or finer. The elevated concentrations in the finer fractions are consistent with results from the literature, which reports that the highest concentrations of arsenic usually occur in the finer particle fractions [6,24]. The elevated arsenic concentrations in the coarser fractions, on the other hand, are most probably caused by aggregation of fine particles, or by very fine particles sticking to coarser grains. The coarsest “clean” (non-aggregated) sediment in the NDRA is the well sorted dune sand of the central dunes and the surficial layer in the southwest and west, with a diameter between 150 and 200 μm, the fraction with the lowest arsenic concentrations in Fig 12. Elsewhere in the NDRA the particles >250 μm (and certainly those >500 μm) in the <2 mm soil are almost exclusively aggregates. This explains the low (<10 μg g^-1^) arsenic concentrations in the west, southwest and center-south in Fig 10 whereas elsewhere in the NDRA the arsenic concentrations are usually >10 μg g^-1^.

### Arsenic in the air

**Fig 13:**
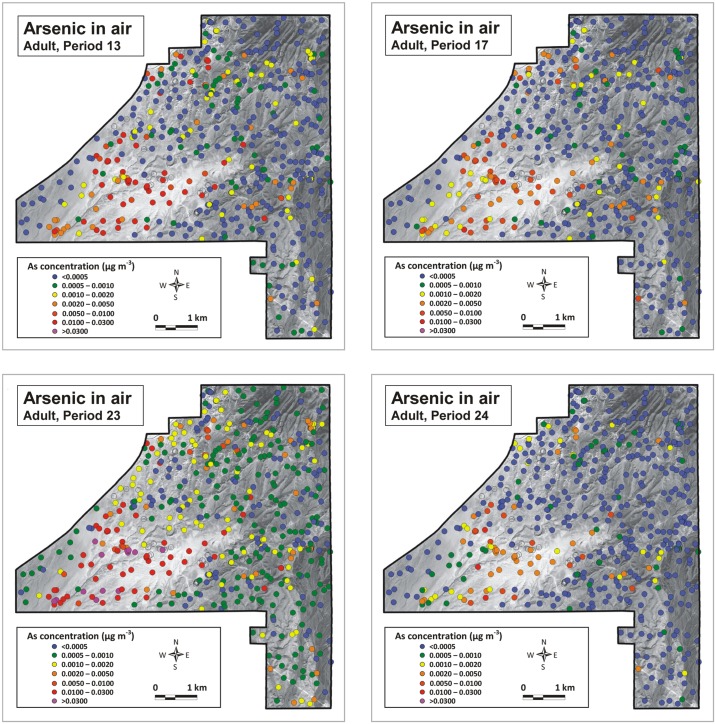
Airborne arsenic concentration at 154.5 cm above the surface (level of nostrils for an average adult American). (A) Period 13: 30 May 2008–12 June 2008; (B) Period 17: 29 July 2008–14 August 2008; (C) Period 23: 21 October 2008–4 November 2008; (D) Period 24: 4 November 2008–18 November 2008. Open circles locate position of bedrock sampling sites. (Background aerial photograph image: Copyright 2009 DigitalGlobe)

**Fig 14:**
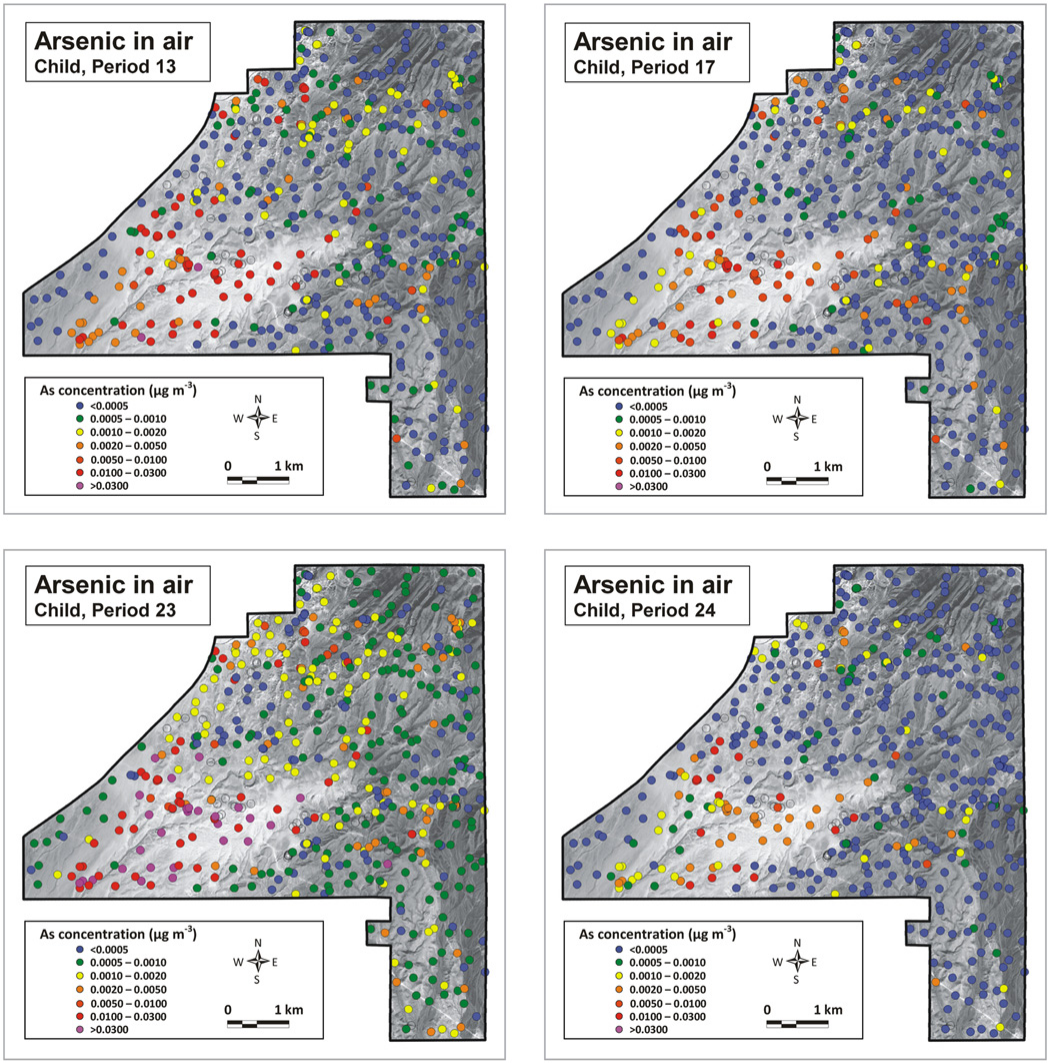
Airborne arsenic concentration at 128.2 cm above the surface (level of nostrils for an average American child of 10 years old). (A) Period 13: 30 May 2008–12 June 2008; (B) Period 17: 29 July 2008–14 August 2008; (C) Period 23: 21 October 2008–4 November 2008; (D) Period 24: 4 November 2008–18 November 2008. Open circles locate position of bedrock sampling sites. (Background aerial photograph image: Copyright 2009 DigitalGlobe)

Using the protocol described above (see section Strategy), airborne arsenic concentrations were calculated for all sampling locations, for the four periods for which dust emission data were available. Results are shown in Figs 13 and 14. Recall that the maps in these figures refer to the arsenic concentration in the entire airborne sediment (Total Suspended Particulates or TSP); not PM10, PM2.5 or other specific fractions. Concentrations for such fractions could not be determined because of the limited amounts of dust collected by the BSNEs. Arsenic concentrations were calculated for two different heights: 154.5 cm and 128.2 cm above the surface, corresponding to the level of the nostrils of an average American adult and an average American child of 10 years old (see [25,26]). Note that no airborne arsenic concentrations were calculated for the sampling points of exposed bedrock because these rocks are not eroded by the wind and also produce no dust when driving over them with a vehicle. These bedrock points are shown as open circles on the airborne arsenic maps in Figs 13 and 14. Airborne arsenic concentration on these locations can be expected to be similar to the airborne arsenic concentration in the direct surroundings.

The airborne arsenic concentrations in the maps for an adult (Fig 13) differ somewhat between measuring periods due to the different wind conditions, but the general pattern is identical for all four periods. By far the highest airborne arsenic concentrations occurred in the dune areas in the center-south and southwest of the NDRA. Elsewhere the arsenic concentrations were low to very low, except in the northwestern corner where concentrations slightly below those in the dunes occurred. The maps for a 10-year-old child are similar, with slightly higher arsenic concentrations due to the closer proximity to the ground, but with the same spatial pattern.

The difference between the airborne and surface patterns (Figs 13 and 14 *versus* Fig 10) is prominent. Areas with high concentrations of arsenic at the surface are not necessarily characterized by high arsenic concentrations in the air and *vice versa*. Although high arsenic concentrations at the surface may result in elevated airborne arsenic concentrations (as shown in the northwest of the NDRA) the aeolian emission rate, quantifying the amount of sand and dust released from the surface during wind erosion appears to be a more important factor. The sandy surfaces in the NDRA, and especially the sand dune sediments (units 1.1 and 1.2 in Fig 3) are by far the most emissive sediments in the NDRA during wind erosion [9]. Despite their low arsenic content in the topsoil these areas experience very high airborne arsenic concentrations during periods of wind erosion. Apart from the dune sands only one other surface type in the NDRA is capable of producing high airborne arsenic concentrations during windy conditions: surface unit 1.5, which is the sandy facies of the yellow sandstone unit. It is responsible for the elevated airborne arsenic concentrations in the northwest. The silt facies of the yellow sandstone unit, represented by surface unit 2.2 (see Fig 3), is very rich in arsenic, but is not very emissive during wind erosion. Therefore the airborne arsenic concentrations in these zones remain low. However, this surface does emit significant arsenic when being driven on by off-road or other vehicles. The patterns in Figs 13 and 14 were constructed for wind erosion; not ORV erosion.

The airborne arsenic maps in Figs 13 and 14 are only for TSP and not for finer fractions such as PM10 or PM2.5, which can be inhaled and are thus of most importance for human health risk assessments. However, the coarser-sized fractions can also be important because those particles are often swallowed. The maps are important because they show the total amount of arsenic present in the air. They also show that, in those parts of the NDRA where emission is low, the airborne arsenic concentrations closely approach the background airborne arsenic concentration in the USA (0.0004 μg m^-3^, see [27,28]). Whether the technique developed in this study also works for PM10 and finer fractions is currently unknown because the (potential) relationship between arsenic content in topsoil and arsenic concentration in airborne dust remains to be explored for these fractions. Future studies are necessary to address this question.

## Conclusions

In the Nellis Dunes Recreation Area elevated arsenic concentrations occur at the surface (both in topsoil and in bedrock) and in the air, especially during periods of wind erosion or during off-road vehicle driving. Excessive concentrations of arsenic at the surface (>1000 μg g^-1^) were measured near the major geologic faults traversing the area. Lower, but still very high arsenic concentrations (>100 μg g^-1^) occur at many other places, usually in outcrops of the yellow sandstone unit. The lowest arsenic concentrations (<10 μg g^-1^) are found in the sand dunes in the center-south and in areas covered by a thin surficial layer of blown dune sand in the west and southwest. Elsewhere in the NDRA the arsenic content at the surface is between 10 and 20 μg g^-1^.

The spatial pattern for arsenic in the air differs strongly from that at the surface. Airborne arsenic concentrations are highest in the dune sand areas despite the low arsenic content in the topsoil. The intense emission of surface sediment during wind erosion is responsible for these high airborne arsenic concentrations. Elsewhere in the NDRA the airborne arsenic concentrations are low, approaching the United States’ background airborne arsenic concentration of 0.0004 μg m^-3^, except in the northwest where elevated airborne arsenic concentrations occur near outcrops of the sandy facies of the yellow sandstone unit.

The elevated arsenic concentrations in the Nellis Dunes Recreation Area are of concern because the two zones of elevated airborne arsenic concentrations (sand dunes in the south and yellow sandstone outcrops in the northwest) coincide with the most visited parts of the NDRA. Approximately 300,000 people visit the NDRA annually [11].

## Supporting Information

S1 Dataset Summary of data in the diagrams and maps. (XLSX)
